# Cyber-victimization—influence of parental rules and impact on mental health among Indian adolescents

**DOI:** 10.3389/fpsyg.2025.1470202

**Published:** 2025-04-30

**Authors:** Rameshbabu Tamarana, Mansi Mathur, Varsha Madhusudan, P. Annapurna Kiranmai

**Affiliations:** ^1^Department of Psychology, Central University of Karnataka, Kalaburagi, India; ^2^Department of Psychology, Christ University, Bangalore, India; ^3^Department of English, Vignan’s (Foundation for Science, Technology & Research), Guntur, India

**Keywords:** cyber-victimization, adolescents, parental rules, mental health, India

## Abstract

**Introduction:**

In the contemporary digital age, cyberspace offers numerous benefits but also presents significant risks, including cyber-victimization. Adolescents, as frequent internet users, are particularly vulnerable to such experiences. This study examines the relationship between parental regulations on internet usage and the incidence of cyber-victimization among Indian adolescents, while also assessing the impact of cyber-victimization on mental health outcomes such as stress, anxiety, and depression.

**Methods:**

A sample of 224 adolescents (Mean age = 16.5 years SD = 2.34) was surveyed using standardized measures of cyber-victimization and mental health.

**Results:**

Multiple linear regression analyses revealed that written-verbal cyber-victimization was a significant predictor of stress (*β* = 0.18, *p* < 0.05), while impersonation, written-verbal cyber-victimization, and online exclusion significantly predicted anxiety (*p* < 0.05). However, none of the cyber-victimization subtypes significantly predicted depression, and the overall model accounted for only 4% of its variance.

**Discussion:**

These findings suggest that while cyber-victimization is linked to stress and anxiety, its influence on depression may be more complex. Furthermore, the Pearson correlation analysis indicated a negligible association between cyber-victimization and parental rules on internet usage (r = 0.039), suggesting that parental regulations alone may not effectively mitigate cyber-victimization risks. Given these findings, interventions focusing on resilience-building, digital literacy, and peer support may be more effective in protecting adolescents from the adverse effects of cyber-victimization. Future research should explore alternative protective factors and preventive strategies to promote adolescent well-being in digital spaces.

## Introduction

Technology has become an indispensable aspect of contemporary existence, completely transforming the way we communicate, entertain ourselves, and educate. The internet has provided several chances for individuals to communicate on a global scale, exchange ideas and information, and get exposure to different perspectives. Adolescents have observed and experienced a significant technological change, relying extensively on the internet and digital platforms for varied needs. However, in the present day, smartphones have become an ever-present companion for adolescents. Adolescents now have access to new spaces for socialising and expressing themselves, thanks to the internet and digital platforms (Abi-Jaoude et al., 2020). Nevertheless, alongside the advantages of technology, there are possible hazards, one of which is cyber-victimization.

### Evolution of cyber-bullying and emergence of cyber-victimisation

Cyberbullying has evolved significantly in its definition and understanding since it emerged as a distinct phenomenon in the digital age. Initially, cyberbullying was defined as intentional and harmful behaviors carried out through electronic means such as computers and mobile devices (Akdeniz and Doğan, 2024). This definition has expanded as researchers have recognized the complex nature of cyberbullying, which can include various forms of aggression such as harassment, impersonation, and exclusion (Álvarez-García et al., 2017). The inconsistency in definitions across studies has contributed to variability in reported prevalence rates, highlighting the need for a standardized understanding of what constitutes cyberbullying (Selkie et al., 2016).

The emergence of cybervictimization, is the deliberate targeting of an individual through negative actions in an online environment, sometimes stemming from adolescent violence and collaboration through technological means (Eroglu et al., 2022). Studies indicate that the prevalence of cybervictimization is alarmingly high, with estimates suggesting that a significant portion of adolescents have experienced some form of online harassment (Akdeniz and Doğan, 2024; Aricak et al., 2008). The relationship between traditional bullying and cyberbullying is also significant; many victims of traditional bullying are also targets of cyberbullying, suggesting a continuation of victimization experiences (Kowalski et al., 2014; van Geel et al., 2014). This overlap indicates that cybervictimization may not only be a standalone issue but also a manifestation of broader patterns of aggression that adolescents face in both online and offline environments (Viau et al., 2019).

### Forms of cyber-victimization

Cyber-victimization includes instances where individuals are subjected to harmful activities via digital platforms, such as cyberbullying, harassment, and exposure to unsuitable content. Due to the growing dependence of adolescents on technology and their frequent use of social media platforms, they are more susceptible to being targeted and victimised online (Suzor et al., 2018). In India, social media trends typically revolve around entertainment, celebrity culture, and local happenings. The strong influence of traditional values and family dynamics in Indian society can be a difficulty for adolescents as they navigate the online world while adhering to cultural standards and meeting parental expectations.

Cyber-victimization possesses various aspects that might have a significant influence on an individual’s mental health. Visual cyber-victimization refers to the unconsented distribution of photos or videos on electronic means to offend or harm an individual. It can manifest in two ways; Teasing/Happy Slapping or Visual Sexual Victimization (Álvarez-García et al., 2017). The individual’s non-consensual dissemination of sexual photos to another person is a violation of privacy. In addition, the perpetrator may engage in blackmail by threatening to distribute the sexual photographs without the victim’s consent until their demands are met (Drouin et al., 2015).

Another aspect to consider is the occurrence of Written Verbal cyber-victimization, which involves an individual being deliberately targeted through phone calls that are unpleasant, threatening, or offensive, as well as through written comments directed against them on the internet. The purpose of this action is to inflict emotional pain on individuals, hence emphasising the occurrence of verbal and psychological abuse in online environments (Nasaescu et al., 2018).

Online exclusion is a discreet yet significant type of cyber-victimization, which refers to the intentional act of excluding an individual from a group or removing them from a group without any wrongdoing on their part, typically on a social network or instant messaging programme. Social isolation and emotional anguish can result from this (Ademiluyi et al., 2022).

Another crucial aspect is Impersonation, which is an individual assuming the identity of the victim either through phone calls or online interactions. This misleading act is usually carried out with the intention of mocking or causing harm to the victim. This can potentially exacerbate their social interactions and perhaps impair their reputation to some degree (Alao and Kohol, 2020). Furthermore, there is an inequality in the availability of internet connection and the level of technological knowledge among various socioeconomic groups and geographical areas (Singh, 2010). This division can heighten the susceptibility of teenagers from marginalized communities to cyber-victimization and the risk of encountering hazardous online information.

### Cyber-victimization among adolescents

Adolescence, a critical stage of development characterised by heightened peer influence, identity formation, and emotional instability, can make individuals more susceptible to the harmful effects of cyber-victimization (Narah, 2020). Adolescents experience social pressure to conform to their peers, which can result in their participating in unsafe online activities, such as disclosing personal information or interacting with unfamiliar individuals. These behaviors increase their vulnerability to cyber-victimization (Lu and Brown, 2023). Increased exposure to social media and online gaming during the COVID-19 pandemic has made adolescents more vulnerable to cyberbullying, with girls being particularly at risk on social media and boys encountering higher risks through online gaming (Marinoni et al., 2024).

Due to the widespread availability of internet access, individuals may encounter detrimental or disturbing content that might have a negative impact on their psychological state (Rahayu et al., 2023). Adolescents who have been subjected to cyber victimization frequently express a sense of social isolation and suffer emotions of shame and embarrassment. These negative feelings can exacerbate their existing mental health problems (Dennehy et al., 2020).

Moreover, being exposed to unsuitable or disturbing material on the internet might result in symptoms of post-traumatic stress disorder, particularly when the information is violent or graphic (Zhu et al., 2023). Cyber-victimization has been linked to stress, suicidal ideation and self-harm, with victims being at a higher risk of experiencing mental health issues such as depression and psychosomatic manifestations (Maurya, 2022; Lin et al., 2020; Kowalski et al., 2014). They may also face social anxiety, relationship difficulties, and decreased academic performance, further contributing to their poor mental well-being (Audrin and Blaya, 2020). Self-oriented personal competencies were the strongest protector against victimization, underscoring the value of fostering resilience, emotional regulation, and coping skills in adolescents to mitigate the psychological toll of online harassment (Zych et al., 2019). Given these potential mental health impacts, parental supervision can play a vital role in managing their children’s online experiences. In India Parent–child relationships are often characterized by a hierarchical, authority-based dynamic with parents playing a significant role in their children’s lives. Research has shown that maladaptive parenting practices, such as neglect or excessive control, can increase the likelihood of cyberbullying and cyber-victimization among adolescents (Achuthan et al., 2022). Equally important is the proactive role of educators in addressing antisocial cyber behaviors, as teachers’ beliefs and conflict resolution skills significantly shape their capacity to manage such behaviors effectively (Guillén-Gámez et al., 2024).

Implementing restrictions on the amount of time spent on screens and establishing clear guidelines for the right use of digital devices can assist teenagers in achieving a harmonious equilibrium between their online and offline activities (Muppalla et al., 2023). Parents have the ability to supervise their children’s online activity and be vigilant in order to detect potential cyber dangers and recognise indications of cyber-victimization. In addition, promoting transparent communication and establishing a secure environment for teenagers to share their online encounters can motivate them to seek assistance when confronted with cyberbullying or exposure to unsuitable material.

### Role of parental support in cybervictimization

According to research on cyber-victimization and parental support, the majority of participants revealed that their parents’ relationship was collaborative. According to almost half of the pupils, neither their fathers nor mothers ever yelled at them or offended them (Iorga et al., 2025). It was found that mothers were more likely to become supportive parents the more educated they were (Iorga et al., 2025). Risk factors that raised the possibility of children bullying or being victimized by their classmates included the use of psychological control and parental neglect (Gómez-Ortiz et al., 2016). Stakeholders emphasized the importance of involving parents, educators, and youth in collaboratively developing and delivering cyberbullying prevention and intervention initiatives (Hendry et al., 2023).

Parental rules exert a significant influence on cyber-victimization experiences among adolescents, with less coercive and more dialogue-based parental engagement acting as protective factors, especially for males. Gender-specific dynamics in cyberbullying necessitate targeted interventions, as females may face distinct pressures (Marinoni et al., 2024).

Effective parental education regarding the risks of digital spaces is crucial, as many parents remain unaware of their children’s activities, including the presence and implications of fake profiles. Striking a balance between monitoring and open dialogue about online safety can empower adolescents to navigate these risks more effectively (Marinoni et al., 2024).

Additionally, it was also found that those who fall victim to cyber-attacks and those who engage in cyber aggression tend to have low levels of self-esteem. Furthermore, their parents exhibit low levels of engagement and acceptance, while displaying high levels of compulsion and imposition towards their children. According to Garaigordobil and Navarro (2022) study, children with authoritarian parents had higher scores in cyber victimization or cyber aggression. Conversely, children with permissive parents reported lower levels of cyber aggression or cyber victimization. The presence of intense family conflict is associated with instances of cyber victimization, whereas negative family communication is associated with reports of insufficient parental support, which in turn is connected to elevated levels of cyberbullying (Buelga et al., 2019).

### Current research objective

With the increasing prevalence of digital interactions, cybervictimization has emerged as a significant concern, particularly among adolescents and young adults. The impact of cybervictimization on psychological well-being can be adverse, particularly among adolescents. The primary source of support and care for adolescents is their parents. So, understanding how parental supervision for internet use by their teen kids can alter the experience and prevalence of cybervictimization, that in turn can help develop effective interventions, school policies, and parental education programs. This research can form the basis for future research to take into account the importance of parental involvement in the context of cyberbullying.

Our research hypotheses that –

1. Cyber-victimization will significantly predict mental health outcomes (stress, anxiety, and depression) among adolescents.
2. There will be a significant positive correlation between different forms of cyber-victimization (impersonation, visual sexual cyber-victimization, written verbal cyber victimization, and online exclusion) and stress.
3. There will be a significant positive correlation between different forms of cyber-victimization and anxiety.
4. There will be a significant positive correlation between different forms of cyber-victimization and depression.
5. That there will be a significant association between cybervictimization and parental rules on internet usage among Indian adolescents

### Theoretical framework

Firstly, Bronfenbrenner’s Ecological Systems Theory is used to understand the interaction between adolescents’ experiences online and their mental health. It examines how various environmental systems influence cyber-victimization and its impact. Evans (2014) states that human development is shaped by various environmental systems that interact. In the context of cyber-victimization, adolescents’ experiences are influenced by multiple levels of ecological systems.

The microsystem includes the people and places closest to the adolescent, such as family, friends, and peers at school. The way parents set internet rules and how peers interact affect the adolescent’s experience with cyber-victimization.

These influences are connected in the mesosystem, where different parts of the adolescent’s life interact. For example, if parents and schools work together by combining parental rules with school digital safety programs, they can better protect adolescents from online harm (Ibrahim et al., 2023).

Beyond these direct influences, the exosystem includes factors that indirectly shape experiences. A parent’s work stress or government policies on digital safety can affect how parents respond to cyber-victimization (Paek et al., 2022).

At a broader level, the macrosystem includes cultural values and societal norms. In India, growing technology use and laws against cybercrime influence how parents regulate internet use and how adolescents perceive online risks (Shah et al., 2016).

Finally, the chronosystem considers how these influences change over time. As technology advances and adolescents grow, their experiences with cyber-victimization also evolve.

The Transactional Model of Stress and Coping (Lazarus and Folkman, 1984) explains how adolescents assess and respond to cyber-victimization. Stress arises from their interaction with the environment and depends on how they evaluate and cope with the situation. First, in primary appraisal, adolescents judge whether cyber-victimization is a serious threat. If they see it as harmful or beyond their control, they may feel overwhelmed and distressed. Next, in secondary appraisal, they consider their coping resources, such as parental support or friendships. Parents who discuss online risks with their children may help more than those who simply restrict internet access (Helsper and Smahel, 2019). Adolescents then choose coping strategies, either problem-focused (e.g., reporting cyberbullying, setting boundaries) or emotion-focused (e.g., suppressing the issue, seeking comfort from others). The effectiveness of these strategies depends on parental guidance and environmental support. In the end, outcomes vary—successful coping builds resilience,

## Method

### Participants

A total of 224 adolescents enrolled from grade 7th to grade 12th participated in this study, the participants were recruited through convenience sampling method. The mean age of the students was 16.57 years, age ranging from 13 to 19 years (SD = 2.34). A total of 118 participants were females and 106 males. Participants were briefed about the study and informed consent was taken before responding to the questionnaires.

### Tools

Cyber Victimization Questionnaire for Adolescents (CYVIC) by Álvarez-García et al. (2017) this questionnaire was used to measure the experience of adolescents having been victimized by cyberspace (mobile phone or internet). It consists of 19 Likert-type response format items (1 = never, 2 = rarely, 3 = sometimes, 4 = always). The items were as “Someone has impersonated me on the internet, posting comments under my name, as if they were me, I have received calls insulting me or making fun of me.” The score ranges from minimum 19 to maximum 76. Higher scores indicate high levels of cyber-victimization. The reliability found in this study was: *α* = 0.662 for Impersonation, α = 0.662 for Visual-Sexual Cybervictimization, α = 0.812 for Written-Verbal Cybervictimization, and α = 0.704 for Online Exclusion. Depression-Anxiety-Stress assessment Scale (DASS-21) by Lovibond and Lovibond (1995). The DAS 21 is a 21 item self-report questionnaire designed to measure the severity of a range of symptoms common to both Depression and Anxiety. In completing the DAS, the individual is required to indicate the presence of a symptom over the previous week. The items were as “I found it hard to wind down, I was aware of dryness of my mouth.” Each item is scored from (did not apply to me at all over the last week) to 3(applied to me very much or most of the time over the past week). Cronbach’s alpha for this scale is 0.80, showing high realiabilty. Parental Rules on Internet Usage Scale—A three-item questionnaire by Barlett and Fennel, used to capture several aspects of parental rules regarding the Internet. Students were asked to indicate their level of agreement with the response on a 1 (completely false) to 5 (completely true) rating scale. The items included, “my parents/guardians have rules about my Internet use,” “my parents/guardians limit my time online,” and “my parents/guardians and I talk about appropriate online behavior.” The cronbach’s alpha for this scale is 0.664.

### Procedure

Permission from schools were obtained to reach out to the students for getting the survey forms filled. Total sample of 224 adolescents was taken for this study, the students were approached from grade 8 to grade 10, they were recruited based on their consent for the participation in the study and their willingness to spare time to fill in the paper pen survey form which took around 15 min approximately. Before administering the questionnaires, the students were debriefed about the study and informed consent was obtained. The administration took place in the month of Februrary and March, 2024.

### Statistical analysis

Analyses were conducted using SPSS software (version 29.0). The correlation analysis was performed to demonstrate the relationship between the scores that the participants obtained from the parental rules on internet usage, CYVIC, and DASS-21 scales. Multiple linear regression was performed to determine the predictability between the scores for the dimensions of cyber victimization, which were predictor variables, and depression, anxiety, stress, which were outcome variables. The results were evaluated at a 95% confidence interval, with a significance level of *p* < 0.05.

## Results

As is seen in Table 1, there was no correlation between parental rules on internet usage and any dimensions of cyber victimization or total cyber victimization scores, depression, anxiety, and stress. There was a low, significant relationship between impersonation and the scores they obtained from the stress (r = 0.16, *p* = 0.02) and anxiety (r = 0.24, *p* = 0.001). There was low, significant positive relationship between written verbal cyber victimization and stress (r = 0.23, *p* = 0.001), anxiety (r = 0.26, *p* = 0.001), and depression (r = 0.17, *p* = 0.01). There was low, significant positive relationship between online exclusion and stress (r = 0.4, *p* = 0.04), anxiety (r = 0.23, *p* = 0.001), and depression (r = 0.17, *p* = 0.01).

**Table 1:** Correlations among the study variables.

Study Variables	1	2	3	4	5	6	7	8	9
1. PR	-	−0.065	0.005	0.034	0.059	0.039	−0.003	0.019	0.076
2. IM	-	-	0.046	0.385^**^	0.219^**^	0.587^**^	0.162^*^	0.244^**^	0.100
3. VSCV	-	-	-	0.066	0.012	0.218^**^	0.075	0.062	0.133
4. WVCV	-	-	-	-	0.246^**^	0.831^**^	0.234^**^	0.257^**^	0.173^*^
5. OE	-	-	-	-	-	0.642^**^	0.143^*^	0.234^**^	0.169^*^
6. CV	-	-	-	-	-	-	0.270^**^	0.338^**^	0.241^**^
7. Stress	-	-	-	-	-	-	-	0.704^**^	0.771^**^
8. Anxiety	-	-	-	-	-	-	-	-	0.634^**^
9. Depression	-	-	-	-	-	-	-	-	-

PR = parental rules on internet usage; IM = impersonation; VSCV = visual cyber victimization; WVCV = written verbal cyber victimization; OE = online exclusion; CV = total cyber victimization. *p < 0.05. **p < 0.01.

Multiple linear regression was designed to predict stress based on their impersonation, visual cyber victimization, written verbal cyber victimization and online exclusion. Table 2 shows that the overall model was significant F(4, 200) = 3.77, *p* < 0.01, *R^2^ = 0*.07, Adjusted *R^2^* = 0.05. The model explains that 5% of variance accounted for by the predictor variables.

**Table 2:** Multiple regression results for stress.

Stress	*B*	95% CI for B		SE B	*β*	*R^2^*	*∆R^2^*
		LL	UL				
Model						0.07	0.05**
Constant	−0.939	−7.331	5.453	3.242			
IM	0.497	−0.535	1.529	0.523	0.071		
VSCV	0.751	−0.974	2.475	0.875	0.059		
WVCV	0.511*	0095	0.927	0.211	0.182*		
OE	0.313	−0.223	0.848	0.272	0.082		

Model = “Enter” method in SPSS Statistics; B = unstandardized regression coefficient; CI = confidence interval; LL = lower limit; UL = upper limit; SE B = standard error of the coefficient; β = standardized coefficient; *R^2^* = coefficient of determination; ∆*R^2^* = adjusted *R^2^*. *p < 0.05. **p < 0.01.

Factors that predict stress include only the written verbal cyber victimization (*β* = 0.18, *p* < 0.05). Specifically, the results suggest that the experiences of written verbal cyber victimization are significantly associated with stress. Regression coefficients and standard errors can be found in Table 2.

Multiple linear regression was designed to predict anxiety based on their impersonation, visual cyber victimization, written verbal cyber victimization and online exclusion. Table 3 shows that the overall model was significant F(4, 200) = 6.63, *p* < 0.001, *R^2^ = 0*.12, Adjusted *R^2^* = 0.10. The model explains that 10% of variance accounted for by the predictor variables. Three variables added statistically significantly to the prediction, *p* < 0.05. Regression coefficients and standard errors can be found in Table 3.

**Table 3:** Multiple regression results for anxiety.

Stress	*B*	95% CI for B		SE B	*β*	*R^2^*	*∆R^2^*
		LL	UL				
Model						0.12	0.10***
Constant	−3.060	−9.287	3.167	3.158			
IM	1.020*	0.015	2.026	0.510	0.145*		
VSCV	0.543	−1.137	2.223	0.852	0.042		
WVCV	0.445*	0.039	0.850	0.205	0.159*		
OE	0.623*	0.101	1.145	0.265	0.163*		

Model = “Enter” method in SPSS Statistics; B = unstandardized regression coefficient; CI = confidence interval; LL = lower limit; UL = upper limit; SE B = standard error of the coefficient; β = standardized coefficient; *R^2^* = coefficient of determination; ∆*R^2^* = adjusted *R^2^*. *p < 0.05. **p < 0.01. ***p < 0.005.

Multiple linear regression was designed to predict depression based on their impersonation, visual cyber victimization, written verbal cyber victimization and online exclusion. Table 4 shows that the overall model was significant F(4, 200) = 3.32, *p* < 0.05, *R^2^ = 0*.06, Adjusted *R^2^* = 0.04. The model explains that only 4% of variance accounted for by the predictor variables. None of the four variables were a significant predictor of depression scores. Regression coefficients and standard errors can be found in Table 4.

**Table 4:** Multiple regression results for depression.

Stress	*B*	95% CI for B		SE B	*β*	*R^2^*	*∆R^2^*
		LL	UL				
Model						0.06	0.04*
Constant	−3.376	−10.787	4.036	3.759			
IM	0.137	−1.060	1.333	0.607	0.017		
VSCV	1.805	−0.194	3.805	1.014	0.122		
WVCV	0.406	−0.076	0.889	0.245	0.126		
OE	0.589	−0.033	1.210	0.315	0.133		

Model = “Enter” method in SPSS Statistics; B = unstandardized regression coefficient; CI = confidence interval; LL = lower limit; UL = upper limit; SE B = standard error of the coefficient; β = standardized coefficient; *R^2^* = coefficient of determination; ∆*R^2^* = adjusted *R^2^*. *p < 0.05. **p < 0.01. ***p < 0.005.

While it is true that one model explains 5% (Table 2) and another (Table 4) explains 4% of the variance, it is essential to recognize that in social and behavioral sciences, it is not uncommon for models to explain relatively small proportions of variance. In the context of our study, even small percentages can be meaningful and provide valuable insights into the factors influencing cyber-victimization and mental health among adolescents. The low variance explained by our models does not diminish the importance of the findings. Instead, it highlights the complexity of the issue and suggests that there are multiple factors at play. Our results contribute to the existing body of knowledge by identifying specific aspects of cyber-victimization that are significantly associated with mental health outcomes such as stress and depression. Additionally, it is important to consider that the explained variance is just one aspect of the model’s overall utility. Our study provides valuable information on the specific relationships between different types of cyber-victimization and mental health outcomes, which can help guide future research.

## Discussion

With the increase in the dependency on internet and electronic devices, the adolescents are vulnerable to be victims of cyber frauds and bullying. The purpose of this research paper is to understand the mental health status of adolescents who are cyber victims and also the association between parental rules on internet usage and cyber-victimization. It was hypothesized that there will be a predictive relationship between cyber-victimization and mental health (Depression, stress & anxiety) among adolescents. The correlation coefficient was calculated between stress, anxiety, depression, and different dimensions of cyber-victimization, namely impersonation, visual sexual cyber-victimization, written verbal cyber victimization, and online exclusion. Starting with stress, the findings reveal a significant positive correlation with impersonation (r = 0.162, *p* < 0.05), and online exclusion (r = 0.143) and written verbal cyber-victimization (r = 0.234, *p* < 0.05), suggesting that higher levels of stress are associated with increased experiences of these forms of cyber-victimization. However, stress showed a weaker association with visual sexual cyber-victimization (r = 0.075) though this correlation was not statistically significant.

Moving to anxiety, the results indicate a significant positive correlation with impersonation (r = 0.244, *p* < 0.05) and written verbal cyber-victimization (r = 0.257, *p* < 0.01). This suggests that individuals experiencing higher levels of anxiety are more likely to report incidents of impersonation, written verbal abuse in online contexts and online exclusion. On the other hand, anxiety shows weaker correlations with visual sexual cyber-victimization (r = 0.062) which is not statistically significant and online exclusion (r = 0.234, *p* < 0.01), with showing a statistically significant association. For depression, the correlations are generally positive but less pronounced compared to stress and anxiety. Depression shows a significant positive correlation with written verbal cyber-victimization (r = 0.173, *p* < 0.05) and online exclusion (r = 0.169). However, there is no significant correlation between depression and impersonation (r = 0.100) & visual sexual cyber-victimization (r = 0.133).

Multiple linear regression models were employed to predict stress, anxiety, and depression based on impersonation, visual cyber victimization, written verbal cyber victimization, and online exclusion. The results revealed interesting patterns. For stress, The overall model was significant (F(4, 200) = 3.77, *p* < 0.01), explaining 5% of the variance in stress. Only written verbal cyber victimization emerged as a significant predictor (*β* = 0.18, *p* < 0.05), suggesting that experiences of written verbal victimization are associated with stress. For Anxiety, The overall model was significant (F(4, 200) = 6.63, *p* < 0.001), explaining 10% of the variance in anxiety. Impersonation, written-verbal cyber-victimization and online exclusion contributed significantly to the prediction (*p* < 0.05), emphasizing their relevance in understanding anxiety. For Depression, The overall model was significant (F(4, 200) = 3.32, *p* < 0.05), but it accounted for only 4% of the variance in depression. None of the four variables were significant predictors of depression scores. In line with the results, there’s another study which found that young individuals who have experienced cyber victimization often report feeling isolated and feelings of shame and embarrassment, which can contribute further to their mental health issues (Dennehy et al., 2020).

The other hypothesis was that there will be a significant association between cyber-victimization and parental rules on internet usage among Indian adolescents. The Pearson correlation coefficient indicated a very weak positive correlation (r = 0.039). However, this value is practically negligible, suggesting that parental rules on internet usage do not significantly predict or explain variations in cyber-victimization. It’s essential to explore other factors beyond parental rules to better understand the occurrence of cyber-victimization among adolescents. By setting limits on screen time and defining appropriate usage of digital devices can help adolescents maintain a healthy balance between their online and offline lives (Muppalla et al., 2023).

Addressing cybervictimization requires a multifaceted approach involving targeted interventions, comprehensive school policies, and proactive parental education. Implementing programs that enhance digital literacy and emotional resilience among youth has proven effective in mitigating the adverse effects of online harassment (Hutson et al., 2017). Schools should adopt holistic anti-cyberbullying policies that include anonymous reporting systems, structured counseling services, and awareness campaigns to foster a supportive environment. Parental engagement plays a crucial role; educating parents on active mediation strategies and promoting open communication can significantly reduce instances of cybervictimization (Wheeler et al., 2023). By integrating these strategies, we can create a safer digital landscape that supports the mental well-being of young individuals. Addressing cybervictimization requires a multifaceted approach involving targeted interventions, comprehensive school policies, and proactive parental education. Implementing programs that enhance digital literacy and emotional resilience among youth has proven effective in mitigating the adverse effects of online harassment (Hutson et al., 2017). Schools should adopt holistic anti-cyberbullying policies that include anonymous reporting systems, structured counseling services, and awareness campaigns to foster a supportive environment. Parental engagement plays a crucial role; educating parents on active mediation strategies and promoting open communication can significantly reduce instances of cybervictimization (Wheeler et al., 2023). By integrating these strategies, we can create a safer digital landscape that supports the mental well-being of young individuals.

## Limitations

The limited sample size reduces the generalizability of the findings, making it difficult to extend conclusions to a broader population. Future studies should consider larger and more diverse samples to enhance external validity.

While parental rules and regulations were considered, a more in-depth investigation into the role of parental mediation, monitoring, and digital literacy could provide a more comprehensive understanding of their impact on cybervictimization and mental health.

The reliance on self-reported data may introduce response bias, as participants might underreport or overreport experiences due to social desirability or recall limitations. Incorporating multiple data sources, such as peer or parental reports, could strengthen the validity of findings.

Other potential buffering factors, such as social support, coping mechanisms, or school-based interventions, may also moderate the impact of cybervictimization on mental health. Future research should explore these additional protective factors to provide a more nuanced perspective.

## Conclusion

Through the results of the study, it is suggestive that cyber-victimization is associated with mental health issues, notably anxiety and stress. As per the findings, impersonation, written-verbal cyber-victimization, and online exclusion raise anxiety levels in adolescents. Also, written verbal cyber-victimization strongly predicts stress. Among the characteristics looked at, the study could not find any significant predictors for depression. The significance of the impact of cyber-victimization as a real issue that might affect adolescents’ psychological health is highlighted by these findings. Cyber-victimization-related stress and anxiety symptoms may be lessened by strategies that minimize written verbal victimization and provide supportive environments both online and offline.

Furthermore, the study indicates that parental rules on internet usage do not appear to have a substantial impact on the incidence of cyber-victimization among adolescents in India. In order to effectively address the issue of cyber-victimization, it appears that more extensive interventions and tactics are required than parental controls. Examples of these include digital literacy programs and supportive school environments. In conclusion, further research is required to examine additional factors and interventions that can better shield adolescents from the negative impacts of online victimization.

## Data Availability

The original contributions presented in the study are included in the article/supplementary material, further inquiries can be directed to the corresponding author.

## References

[ref1] Abi-Jaoude E. Naylor K. T. Pignatiello A. (2020). Smartphones, social media use and youth mental health. Canad. Med. Assoc. J. 192, E136–E141. doi: 10.1503/cmaj.190434, PMID: 32041697 PMC7012622

[ref2] Achuthan K. Muthupalani S. Kolil V. K. Madathil K. C. (2022). Theoretical perspectives of parental influence on adolescent cyber behaviour: a bi-national Instagram-based study. Heliyon 8:e11813. doi: 10.1016/j.heliyon.2022.e11813, PMID: 36451757 PMC9703604

[ref3] Ademiluyi A. Li C. Park A. (2022). Implications and preventions of cyberbullying and social exclusion in social media: systematic review. JMIR Form. Res. 6:e30286. doi: 10.2196/30286, PMID: 34982712 PMC8767467

[ref4] Akdeniz B. Doğan A. (2024). Cyberbullying: definition, prevalence, effects, risk and protective factors. Psikiyatride Güncel Yaklaşımlar 16, 425–438. doi: 10.18863/pgy.1325195

[ref5] Alao S. Kohol B. (2020). The influence of cyber-impersonation on psycho-social adjustment of public university students in north central states of Nigeria. IOSR J. Res. Method Educ. 10, 34–39. doi: 10.9790/7388-1001063439

[ref6] Álvarez-García D. Núñez J. C. Barreiro-Collazo A. García T. (2017). Validation of the Cybervictimization questionnaire (CYVIC) for adolescents. Comput. Hum. Behav. 70, 270–281. doi: 10.1016/j.chb.2017.01.007

[ref7] Aricak T. Siyahhan S. Uzunhasanoglu A. Saribeyoglu S. Ciplak S. Yilmaz N.. (2008). Cyberbullying among Turkish adolescents. Cyberpsychol. Behav. 11, 253–261. doi: 10.1089/cpb.2007.0016, PMID: 18537493

[ref8] Audrin C. Blaya C. (2020). Psychological well-being in a connected world: the impact of Cybervictimization in Children’s and young People’s life in France. Front. Psychol. 11, 2–3. doi: 10.3389/fpsyg.2020.01427, PMID: 32765342 PMC7380249

[ref9] Buelga S. Martínez-Ferrer B. Cava M.-J. Ortega-Barón J. (2019). Psychometric properties of the CYBVICS cyber-victimization scale and its relationship with psychosocial variables. Soc. Sci. 8:13. doi: 10.3390/socsci8010013

[ref10] Dennehy R. Meaney S. Cronin M. Arensman E. (2020). The psychosocial impacts of cybervictimisation and barriers to seeking social support: young people’s perspectives. Child Youth Serv. Rev. 111:104872. doi: 10.1016/j.childyouth.2020.104872

[ref11] Drouin M. Ross J. Tobin E. (2015). Sexting: a new, digital vehicle for intimate partner aggression? Comput. Hum. Behav. 50, 197–204. doi: 10.1016/j.chb.2015.04.001

[ref12] Eroglu Y. Peker A. Cengiz S. (2022). Cyber victimization and well-being in adolescents: the sequential mediation role of forgiveness and coping with cyberbullying. Front. Psychol. 13:2. doi: 10.3389/fpsyg.2022.819049, PMID: 36467151 PMC9716218

[ref800] Evans O. Q. (2014). Bronfenbrenner’s Ecological Systems Theory. Simply Psychology, https://www.simplypsychology.org/bronfenbrenner.html

[ref14] Garaigordobil M. Navarro R. (2022). Parenting styles and self-esteem in adolescent Cybervictims and Cyberaggressors: self-esteem as a mediator variable. Children 9:1795. doi: 10.3390/children9121795, PMID: 36553238 PMC9777360

[ref15] Guillén-Gámez F. D. Colomo-Magaña E. Doddanavar I. Ortiz-Padilla M. (2024). Incidence of factors in teaching actions to manage antisocial Cyberbehaviour. TechTrends 69, 111–124. doi: 10.1007/s11528-024-01029-x, PMID: 40232369

[ref9001] Gomez-Ortiz O. Romera E. M. Ortega-Ruiz R. (2016). Parenting styles and bullying. The mediating role of parental psychological aggression and physical punishment. Child Abuse & Neglect, 51, 132–143. doi: 10.1016/j.chiabu.2015.10.025 26598076

[ref16] Helsper E. J. Smahel D. (2019). Excessive internet use by young Europeans: psychological vulnerability and digital literacy? Inf. Commun. Soc. 23, 1255–1273. doi: 10.1080/1369118x.2018.1563203, PMID: 40101104

[ref17] Hendry B. P. Hellsten L. M. McIntyre L. J. Smith B. R. R. (2023). Recommendations for cyberbullying prevention and intervention: a Western Canadian perspective from key stakeholders. Front. Psychol. 14:6–9. doi: 10.3389/fpsyg.2023.1067484, PMID: 36960003 PMC10027701

[ref18] Hutson E. Kelly S. Militello L. K. (2017). Systematic review of cyberbullying interventions for youth and parents with implications for evidence-based practice. Worldviews Evid.-Based Nurs. 15, 72–79. doi: 10.1111/wvn.12257, PMID: 28859246 PMC8074991

[ref19] Ibrahim K. Oshodi O. S. Osuizugbo I. C. Owusu E. K. Ibrahim A. Shakantu K. K. (2023). The usage of social media for learning and teaching in the built environment: a perspective from students. Int. J. Constr. Manag. 24, 902–910. doi: 10.1080/15623599.2023.2239482, PMID: 40101104

[ref20] Iorga M. Pop L. M. Croitoru I. Hanganu E. Anton-Păduraru D. T. (2025). 10.3390/bs12110457 PMC9687515 36421753

[ref21] Kowalski R. M. Giumetti G. W. Schroeder A. N. Lattanner M. R. (2014). Bullying in the digital age: a critical review and meta-analysis of cyberbullying research among youth. Psychol. Bull. 140, 1073–1137. doi: 10.1037/a0035618, PMID: 24512111

[ref22] Lazarus R. Folkman S. (1984). Stress, appraisal, and coping. New York: Springer.

[ref23] Lin L. Liu J. Cao X. Wen S. Xu J. Xue Z.. (2020). Internet addiction mediates the association between cyber victimization and psychological and physical symptoms:moderation by physical exercise. BMC Psychiatry 20:144. doi: 10.1186/s12888-020-02548-6, PMID: 32245443 PMC7118978

[ref24] Lu K. Brown C. (2023). Susceptibility to peer pressure among adolescents: biological, demographic, and peer-related determinants. J. Stud. Res. 11, 1–6. doi: 10.47611/jsr.v11i1.1576

[ref9002] Lovibond S. H. Lovibond P. F. (1995). Depression Anxiety Stress Scales. PsycTESTS Dataset. doi: 10.1037/t01004-000 7726811

[ref25] Marinoni C. Rizzo M. Zanetti M. A. (2024). Fake profiles and time spent online during the COVID 19 pandemic: a real risk for cyberbullying? Curr. Psychol. 43, 26639–26647. doi: 10.1007/s12144-024-05979-6

[ref26] Maurya C. (2022). The effects of cyberbullying victimization on depression and suicidal ideation among adolescents and young adults: a three year cohort study from India. BMC Psychiatry 22:599. doi: 10.1186/s12888-022-04238-x, PMID: 36085004 PMC9461154

[ref27] Muppalla S. K. Vuppalapati S. Pulliahgaru A. R. Sreenivasulu H. (2023). Effects of excessive screen time on child development: an updated review and strategies for management. Cureus 15, 1–5. doi: 10.7759/cureus.40608, PMID: 37476119 PMC10353947

[ref28] Narah B. (2020). Needs and problems of adolescence period: proper guidance and counselling in the field of their future LIFE construction. Int. J. Adv. Res. 8, 169–173. doi: 10.21474/ijar01/10612

[ref29] Nasaescu E. Marín-López I. Llorent V. J. Ortega-Ruiz R. Zych I. (2018). Abuse of technology in adolescence and its relation to social and emotional competencies, emotions in online communication, and bullying. Comput. Hum. Behav. 88, 114–120. doi: 10.1016/j.chb.2018.06.036

[ref30] Paek S. Y. Lee J. Choi Y. (2022). The impact of parental monitoring on cyberbullying victimization in the COVID-19 era. Soc. Sci. Q. 103, 294–305. doi: 10.1111/ssqu.13134, PMID: 35602177 PMC9115449

[ref31] Rahayu N. I. Falimu N. Rumondor N. P. Kurniawati N. H. Aziz A. M. (2023). Promoting mental health in the digital age: exploring the effects of social media use on Psyhcological well-being. West Sci. Interdiscip. Stud. 1, 239–247. doi: 10.58812/wsis.v1i6.95

[ref32] Selkie E. M. Fales J. L. Moreno M. A. (2016). Cyberbullying prevalence among US middle and high school-aged adolescents: a systematic review and quality assessment. J. Adolesc. Health 58, 125–133. doi: 10.1016/j.jadohealth.2015.09.026, PMID: 26576821 PMC4724486

[ref33] Shah T. Yavari A. Mitra K. Saguna S. Jayaraman P. P. Rabhi F.. (2016). Remote health care cyber-physical system: quality of service (QoS) challenges and opportunities. IET Cyber Phys. Syst. 1, 40–48. doi: 10.1049/iet-cps.2016.0023

[ref34] Singh S. (2010). Digital Divide in India. Int. J. Innov. Digit. Econ. 1, 1–24. doi: 10.4018/jide.2010040101

[ref35] Suzor N. Dragiewicz M. Harris B. Gillett R. Burgess J. Van Geelen T. (2018). Human rights by design: the responsibilities of social media platforms to address gender-based violence online. Policy Internet 11, 84–103. doi: 10.1002/poi3.185, PMID: 40232040

[ref36] van Geel M. Vedder P. Tanilon J. (2014). Relationship between peer victimization, cyberbullying, and suicide in children and adolescents. JAMA Pediatr. 168:435. doi: 10.1001/jamapediatrics.2013.4143, PMID: 24615300

[ref37] Viau S.-J. Denault A.-S. Dionne G. Brendgen M. Geoffroy M.-C. Côté S. M.. (2019). Joint trajectories of peer cyber and traditional victimization in adolescence: a look at risk factors. J. Early Adolesc. 40, 936–965. doi: 10.1177/0272431619880339, PMID: 40225811

[ref9003] Wheeler B. Baumel K. Hall D. Silva Y. (2023). U.S. parents’ intentions to use antibullying apps: Insights from a comprehensive model. Heliyon, 9, 1–14. doi: 10.1016/j.heliyon.2023.e19630 PMC10558870 37809431

[ref38] Zhu X. Zhu W. Yu W. (2023). Effects of media exposure on PTSD symptoms in college students during the COVID-19 outbreak. Front. Public Health 11, 8–10. doi: 10.3389/fpubh.2023.1050759, PMID: 37228721 PMC10203595

[ref39] Zych I. Farrington D. P. Ttofi M. M. (2019). Protective factors against bullying and cyberbullying: a systematic review of meta-analyses. Aggress. Violent Behav. 45, 4–19. doi: 10.1016/j.avb.2018.06.008, PMID: 40231187

